# Life-course socioeconomic circumstances in acute, chronic and disabling pain among young employees: a double suffering

**DOI:** 10.1177/14034948211062314

**Published:** 2021-12-29

**Authors:** Pi Fagerlund, Jatta Salmela, Olli Pietiläinen, Aino Salonsalmi, Ossi Rahkonen, Tea Lallukka

**Affiliations:** Department of Public Health, University of Helsinki, Helsinki, Finland

**Keywords:** Pain, chronic pain, occupations, socioeconomic factors, education, disabled persons, adverse childhood experiences

## Abstract

**Background:**

Pain is known to be socioeconomically patterned and associated with disability. However, knowledge is scarce concerning life-course socioeconomic circumstances and pain among young adults. Our aim was to examine the associations of childhood and current socioeconomic circumstances with acute pain and chronic pain with low and high disability levels among young Finnish municipal employees.

**Methods:**

We analysed questionnaire data retrieved from the Young Helsinki Health Study (*n*=4683) covering 18–39-year-old employees of the City of Helsinki, Finland. We included multiple indicators of childhood and current socioeconomic circumstances and examined their associations with acute pain and with chronic pain with low and high disability levels. The level of chronic pain-related disability was assessed by the chronic pain grade questionnaire. Multinomial logistic regression analyses were conducted with stepwise adjustments for sociodemographic, socioeconomic and health-related covariates.

**Results:**

Childhood and current socioeconomic disadvantage were associated with acute and chronic pain, particularly with chronic pain with high disability level. The strongest associations after adjustments for covariates remained between chronic pain with high disability level and low educational level (odds ratio (OR) 3.38, 95% confidence interval (CI) 2.18–5.24), manual occupation (OR 3.75, 95% CI 1.92–7.34) and experiencing frequent economic difficulties (OR 3.07, 95% CI 2.00–4.70).

**Conclusions:**

**Pain is a common complaint that contributes to disability among young employees, particularly the most socioeconomically vulnerable. There is a socioeconomic gradient in both pain chronicity and the level of chronic pain-related disability. Life-course socioeconomic factors should be considered in pain-preventing strategies and in clinical practice.**

## Introduction

Pain is one of the leading causes of disability globally [1] and Finland is not exempt from the burdens that pain poses on individuals and society. Nearly one-third of aging female Finnish employees report chronic pain [2]. Chronic pain is, furthermore, associated with disability retirement due to musculoskeletal and mental disorders, the two most common reasons for receiving disability pension in Finland [3].

Pain is subjective and has both biological and psychosocial aspects, the latter being important particularly in chronic pain conditions [4]. Pain chronicity is a central concept when considering long-term implications of pain. The level of pain-related disability is another important indicator as it reflects both pain intensity and pain interference and has implications for quality of life [5,6].

Current evidence suggests an inverse relationship between socioeconomic position (SEP) and chronic pain [2,7]. SEP is reflected by a multitude of material and social factors, and simultaneous consideration of several measures is needed to capture different aspects of SEP and their changes over the life course [8]. Still, most existing studies on SEP and pain use only single or few measures of SEP [7,9].

Pain is common among young people. Of 18–25-year-old British adults, 67% reported pain within the past 6 months and 3% reported severely disabling chronic pain [10]. However, studies on SEP and pain have mostly been population based or focused on older individuals [2, 7, 9]. Knowledge is hence limited regarding chronic pain among young adults, for whom pain and disability can be decisive for their future life trajectory and career advancement [10]. An inverse association between SEP before midlife and subsequent chronic pain has been observed, suggesting that disadvantaged SEP in early adulthood is a risk factor for later chronic pain [11]. However, the significance of life-course SEP for pain among young adults needs further exploration.

The aim of this study was to quantify the prevalence of pain and examine whether life-course socioeconomic factors are associated with pain prevalence, pain chronicity and chronic pain-related disability in a cohort of young Finnish municipal employees. We examined the associations of multiple childhood and current socioeconomic circumstances with (a) acute pain, (b) chronic pain with low disability level (CPLD) and (c) chronic pain with high disability level (CPHD). Sociodemographic factors such as gender, age and ethnicity have been linked to pain, and differences in health behaviours are known mediators of socioeconomic differences in health [12,13]. Therefore, we considered the contribution of these factors to the associations.

## Methods

### Study population

**Figure 1.**
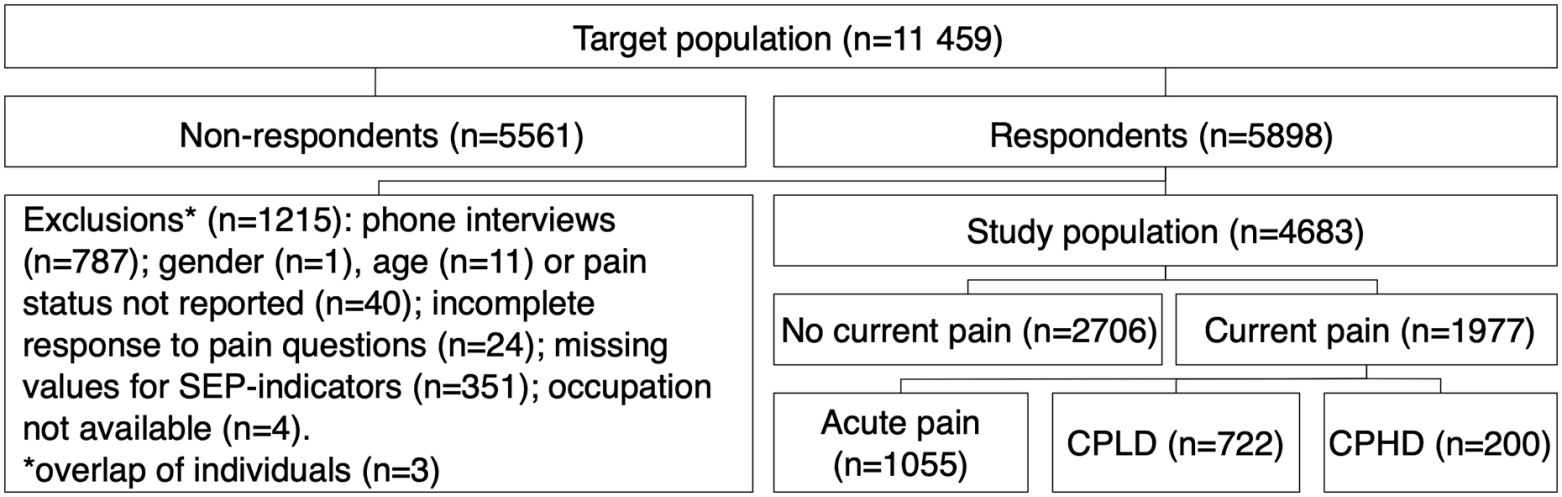
Participant selection and classification of pain status. CPLD: chronic pain with low disability level; CPHD: chronic pain with high disability level.

Our data were derived in 2017 from the Young Helsinki Health Study cohort, which follows the health and wellbeing of initially 18–39-year-old employees of the City of Helsinki [14]. The majority (76%) of employees were women. The main criterion for inclusion to the target population was being a current employee of the City of Helsinki, born in 1978 or later. Further criteria were having a working relationship with a duration of more than 4 months prior to the survey and a work contract with the City of Helsinki, with at least 50% regular working hours a week. The final target population consisted of 11,459 employees [14]. Data were collected either online (58%) or mailed (29%) questionnaires or telephone interviews (13%). An online questionnaire was sent to the employees’ office email. Questionnaires were sent by mail to employees lacking an office email or as a reminder to those not responding to the online survey. Those not responding despite reminders were selected for phone interviews, provided their phone numbers were available. The final response rate was 51.5% (*n*=5898). Certain exclusion criteria were applied to the respondents (Figure 1). Respondents answering by phone interview (*n*=787) were excluded because this short interview included only some of the variables of interest in this study. After exclusion, 4683 respondents were included in the study sample.

### Measures of socioeconomic circumstances

#### Childhood SEP indicators

Parental educational level and childhood economic difficulties were used as indicators of childhood SEP. Respondents reported the highest educational level of each parent on a four-level scale (1: middle school or less; 2: vocational school, college or equivalent; 3: upper secondary school; 4: university degree), and the highest educational level of either parent was chosen. Information on childhood economic difficulties was obtained by a yes/no question about whether the respondent had experienced economic difficulties in the family before the age of 16 years.

#### Current SEP indicators

Current SEP was assessed by the respondent’s own educational level, occupational class, housing tenure, household income, household wealth and current economic difficulties. The respondents’ highest educational level was divided into high (master’s degree or higher), intermediate (bachelor’s degree) or low (upper secondary school or lower). Based on occupation, respondents were classified as manual workers, routine non-manual workers, semi-professionals and professionals. Housing tenure status was divided into owner-occupiers and renters or other. Household income was equivalised by household size by dividing the typical monthly net household income (10-level scale) by a weight factor according to the modified Organisation for Economic Co-operation and Development (OECD) equivalence scale: the respondent received the value of 1.0, other adults 0.5 and children 0.3, and these values were added together [15]. The equivalised household income was further divided into gender-specific quartiles that were combined into a common variable for both genders. Household wealth was divided into a high (⩾ €100k), intermediate ( €10k–99,999) and low (< €10k) wealth. Current economic difficulties were assessed by two questions: (a) ‘How often do you have enough money to afford the kind of food or clothing you/your family should have?’ and (b) ‘How much difficulty do you have in meeting the payment of bills?’. Response categories ranged from ‘never’ to ‘always’ and ‘very great’ to ‘very little or none’. A summed score was formed and divided into three categories: no, occasional, and frequent difficulties [16].

### Measures of pain

#### Pain prevalence and chronicity

The prevalence of current pain was assessed by the question ‘Are you suffering from any pains or aches right now?’ (‘no/yes’). Respondents reporting current pain were asked whether the pain had lasted for a shorter or longer time than 3 months. Pain with a duration of less than 3 months was defined as acute, whereas pain with a duration of 3 months or longer was defined as chronic [17].

#### Level of chronic pain-related disability

Participants reporting current pain were invited to complete the chronic pain grade questionnaire (CPG), a tool for the assessment of chronic pain-related disability [5]. The CPG consists of seven questions covering the intensity, persistence and interference of chronic pain with daily activities, working capacity and social life during the past 6 months. A disability score ranging from 0 to 6 was calculated based on answers provided to the seven questions. The disability score was further dichotomised into CPLD (score 0–2) and CPHD (score 3–6) [5].

### Covariates

#### Sociodemographic factors

Sociodemographic factors considered were gender, age, immigrant background, marital status and employment status. Gender was reported as male or female. Age was calculated from the birth year and dichotomised into less than 30 years and 30–39 years. Respondents who were either themselves or had one or more parent born outside Finland were classified as having an immigrant background. Marital status was divided into married or cohabiting and other. Although the assumption was that all respondents were employed at the time of data collection, we included a variable for employment status among the sociodemographic factors. This was done to distinguish respondents who currently worked from respondents who were temporarily outside the labour market (e.g. studying, on parental leave or on long-term sickness absence), because the working status might have changed between the time of inclusion and submission of responses. Respondents in full-time or part-time work were considered as working, and in other cases were considered as not working.

#### Health-related factors

We included a range of health-related covariates due to their potential confounding or mediating effect between pain outcomes and socioeconomic variables [2,18]. These were binge drinking, nicotine use, leisure-time physical activity (LTPA), body mass index (BMI), insomnia and mental health. All covariates were dichotomised, except for physical activity, which was divided into three levels. Respondents reporting binge drinking (⩾6 servings) weekly or more frequently were classified as binge drinkers. Respondents who used cigarettes, snuff or electronic cigarettes daily or sporadically were classified as nicotine users. LTPA was measured in metabolic equivalent hours per week, based on the self-reported weekly amount and intensity of LTPA, and it was divided into low, intermediate and high activity levels. BMI (weight/height^2^) was calculated based on self-reported values and dichotomised with a cut-off value of 30 kg/m^2^. Insomnia was defined as reporting one or more symptom of sleeping difficulties in more than 14 nights per month [19]. Mental health was scored based on the 12-item general health questionnaire (GHQ-12) and dichotomised into not having (0–2 points) and having mental health problems (⩾3 points) [20]. In cases with missing values in a covariate (range 0.0–1.0%, and for binge drinking 3.6%), the respondent was included in the comparison group of that variable.

### Statistical methods

#### Correlation and gender interaction analyses

Correlation analyses (Spearman’s correlation) were performed for SEP variables to examine the level of correlation and multicollinearity (Supplenental Table I). There was a strong correlation between a participant’s own educational level and occupational class (women *r*=0.797, men *r*=0.723), and a moderate correlation between wealth and housing tenure (women *r*=0.587, men *r*=0.577), while all other correlations were substantially lower. No indication for multicollinearity was found (the variance inflation factor was 1.1–2.7 for women and 1.1–2.1 for men). However, to avoid over-adjustment due to correlation between variables, we did not adjust the regression analyses for current SEP indicators.

Female employees accounted for 79.8% (*n*=3736) of the study population and male employees for 20.2% (*n*=947). Due to the low number of male respondents, a gender-stratified approach was not suitable for multinomial logistic regression analyses. To ensure the suitability of a non-gender-stratified analysis, gender interaction analyses were performed for the socioeconomic variables and the pain outcomes. Diverging associations between genders were observed for the prevalence of pain with housing tenure status (*P*=0.02) and with wealth (*P*=0.02), likewise for the level of pain-related disability with occupational class (*P*=0.02). Beyond these few findings, the associations were similar between genders, and the overall gender interaction was minor. Thus, all respondents were hereafter handled as one sample, and gender was adjusted for.

#### Multinomial regression analyses

To examine the associations of measures of SEP with pain outcomes, we performed a series of multinomial logistic regression analyses for which we calculated odds ratios (ORs) and their 95% confidence intervals (CIs). The models were built in three steps. In model 1, each childhood and current socioeconomic variable was adjusted for gender and age. In model 2, model 1 was further adjusted for childhood SEP indicators. In model 3, model 2 was adjusted for sociodemographic indicators (marital status, immigrant background and employment status) and health indicators (binge drinking, nicotine use, LTPA, BMI, insomnia and mental health). Respondents reporting no pain and respondents with the most advantaged SEPs were considered as reference groups. All statistical analyses were conducted using IBM SPSS version 25.

### Ethical considerations

The study plan obtained approval from the City of Helsinki and a positive statement by the research ethics committee of the Faculty of Medicine, University of Helsinki, Finland.

## Results

### Descriptive results

Current pain was reported by 42.2% of the young employees. Acute pain was reported by 22.5%, CPLD by 15.4% and CPHD by 4.3% of the respondents. The prevalence of pain was consistently higher among women than among men (*P*<0.001) (Table I). Furthermore, individuals with childhood or present socioeconomic disadvantage were overrepresented among participants who reported acute or chronic pain, particularly CPHD (Table II). For example, individuals with a low educational level comprised 33.5% of all respondents, whereas the corresponding share was 52.0% among those reporting CPHD. The prevalence of mental health problems was 35.0% in the entire study population but as high as 63.5% among individuals with CPHD. Individuals reporting insomnia, BMI of 30 kg/m^2^ or greater and nicotine use were also more likely to report CPHD, whereas highly physically active individuals were underrepresented in all pain categories (Tables I and II).

**Table I.** Participant characteristics by gender (*n*=4683).

	Men (%)	Women (%)	Total (%)	Total (*n*)	*P* value
Pain outcome					<0.001
No pain	65	55.9	57.8	2706	
Acute pain	17.2	23.9	22.5	1055	
CPLD	14.9	15.6	15.4	722	
CPHD	2.9	4.6	4.3	200	
Parental education level					0.080
Higher education	45.8	42.5	43.2	2023	
Upper secondary school	13.2	12.4	12.5	587	
Vocational school	31.5	35.9	35	1641	
Elementary school	9.5	9.2	9.2	432	
Childhood economic difficulties					0.139
No	76.6	78.8	78.3	3668	
Yes	23.4	21.2	21.7	1015	
Own education level					<0.001
High	28.5	29.8	29.5	1383	
Intermediate	29.4	38.9	37	1732	
Low	42.1	31.3	33.5	1568	
Occupational class					<0.001
Professional	30.8	26.9	27.7	1298	
Semi-professional	29.9	43	40.3	1888	
Routine non-manual employee	24.8	27.3	26.8	1256	
Manual worker	14.5	2.8	5.1	241	
Housing tenure					0.569
Owner-occupier	43.6	42.6	42.8	2004	
Renter (or other)	56.4	57.4	57.2	2679	
Income level					0.008
4th Quartile (highest)	24.3	24.1	24.1	1129	
3rd Quartile	29.4	25.4	26.2	1227	
2nd Quartile	20.2	24.9	24	1123	
1st Quartile (lowest)	26.2	25.6	25.7	1204	
Wealth (€)					<0.001
⩾100k	24.5	24.5	24.5	1148	
10k–99,999	45.9	39	40.4	1893	
<10k	29.6	36.5	35.1	1642	
Economic difficulties					0.057
No difficulties	48	45	45.6	2136	
Occasional difficulties	43.5	44.2	44.1	2063	
Frequent difficulties	8.4	10.8	10.3	484	
Age					0.004
<30 Years	28.1	32.9	31.9	1496	
⩾30 Years	71.9	67.1	68.1	3187	
Immigrant background					0.034
No	89.8	91.9	91.5	4284	
Yes	10.2	8.1	8.5	399	
Marital status					<0.001
Married or co-habiting	71.4	65.2	66.5	3112	
Other or missing	28.6	34.8	33.5	1571	
Employment status					<0.001
Working	97.1	88.2	90	4216	
Not working	2.9	11.8	10	467	
Binge drinking					<0.001
No binge drinking or missing	83.8	96.2	93.7	4388	
Weekly or more frequently	16.2	3.8	6.3	295	
Nicotine use					<0.001
No use or missing	60.7	75.4	72.4	3392	
Daily or sporadically	39.3	24.6	27.6	1291	
Physical activity					<0.001
High or missing	70.2	59.6	61.7	2891	
Intermediate	19.7	31.3	28.9	1355	
Low	10	9.2	9.3	437	
BMI					0.686
<30 kg/m^2^ or missing	86.2	85.7	85.8	4016	
⩾30 kg/m^2^	13.8	14.3	14.2	667	
Insomnia					<0.001
No insomnia or missing	76.6	66.9	68.8	3223	
Insomnia	23.4	33.1	31.2	1460	
Mental health (GHQ)					<0.001
No mental health problems or missing	73.2	62.9	65	3043	
Mental health problems	26.8	37.1	35	1640	

BMI: body mass index; CPLD: chronic pain with low disability level; CPHD: chronic pain with high disability level; GHQ: general health questionnaire.

*P* values calculated using the chi-square test of independence.

**Table II.** Variables by pain outcomes, *P* values.

	No pain (%)	Acute pain (%)	CPLD (%)	CPHD (%)	*P* value
	(*n*=2706)	(*n*=1055)	(*n*=722)	(*n*=200)	
Parental education level					
Higher education	45.4	38.7	43.8	35	0.001
Upper secondary school	12.7	12.4	11.9	13	
Vocational school	33.9	38.2	33.8	39	
Elementary school	8	10.7	10.5	13	
Childhood economic difficulties					<0.001
No	81.5	77	72.7	62.5	
Yes	18.5	23	27.3	37.5	
Own education level					<0.001
High	32.8	25.2	26.9	18	
Intermediate	37.4	37.2	37.1	30	
Low	29.8	37.6	36	52	
Occupational class					<0.001
Professiona	29.8	24.5	27.1	18.5	
Semi-professional	40.8	40.3	39.3	37.5	
Routine non-manual employee	24.7	30	27.4	36	
Manual worker	4.7	5.1	6.1	8	
Housing tenure					<0.001
Owner occupier	44.6	39.1	43.8	33.5	
Renter (or other)	55.4	60.9	56.2	66.5	
Income level					0.001
4th Quartile (highest)	25.5	23.3	22.2	16	
3rd Quartile	26.9	25.4	25.8	22	
2nd Quartile	23	26.5	24.5	22	
1st Quartile (lowest)	24.5	24.7	27.6	40	
Wealth (€)					<0.001
⩾100k	27.3	21.3	21.1	16	
10k–99,999	40.7	40.3	41.7	32.5	
<10k	32	38.4	37.3	51.5	
Economic difficulties					<0.001
No difficulties	51	40.9	36.6	30.5	
Occasional difficulties	40.9	47.2	51.4	43	
Frequent difficulties	8.1	11.8	12	26.5	
Gender					<0.001
Men	22.8	15.5	19.5	13.5	
Women	77.2	84.5	80.5	86.5	
Age					0.020
<30 Years	32.7	33	30.1	23	
⩾30 Years	67.3	67	69.9	77	
Immigrant background					0.001
No	92	92.4	90.2	84.5	
Yes	8	7.6	9.8	15.5	
Marital status					0.188
Married or co-habiting	67.1	66.9	65.2	60	
Other or missing	32.9	33.1	34.8	40	
Working status					0.298
Working	89.6	91.4	89.2	91.5	
Not working	10.4	8.6	10.8	8.5	
Binge drinking					0.611
No binge drinking or missing	93.4	93.8	94.7	93.5	
Weekly or more frequently	6.6	6.2	5.3	6.5	
Nicotine use					0.001
No use or missing	74.4	70.2	70.4	64.5	
Daily or sporadically	25.6	29.8	29.6	35.5	
Physical activity					<0.001
High or missing	64.3	59.9	56.6	55.5	
Intermediate	27.2	31	30.9	34	
Low	8.5	9.1	12.5	10.5	
BMI					<0.001
<30 kg/m^2^ or missing	87.6	84.5	83.1	77.5	
⩾30 kg/m^2^	12.4	15.5	16.9	22.5	
Insomnia					<0.001
No insomnia or missing	76.2	62.7	56.2	47	
Insomnia	23.8	37.3	43.8	53	
Mental health (GHQ)					<0.001
No mental health problems or missing	71.7	57.8	58.3	36.5	
Mental health problems	28.3	42.2	41.7	63.5	

BMI: body mass index; CPLD: chronic pain with low disability level; CPHD: chronic pain with high disability level; GHQ: general health questionnaire.

*P* values calculated using the chi-square test of independence.

### Childhood and adult SEP inequalities in pain

Childhood SEP was associated with pain outcomes (Table III). Regarding parental education, we found SEP differences in all three pain categories after gender and age adjustment (model 1). This association was strongest for CPHD (OR 1.98, 95% CI 1.23–3.19) and decreased with higher parental educational level. Concerning childhood economic difficulties, a similar pattern was found, with a consistently higher prevalence of pain in economically disadvantaged individuals, particularly for CPHD (OR 2.62, 95% CI 1.93–3.55).

**Table III.** Associations of childhood and current socioeconomic circumstances with acute pain, CPLD and CPHD, based on multinomial logistic regression analyses.

	Model 1			Model 2			Model 3		
	Acute pain	CPLD	CPHD	Acute pain	CPLD	CPHD	Acute pain	CPLD	CPHD
	OR (95% CI)	OR (95% CI)	OR (95% CI)	OR (95% CI)	OR (95% CI)	OR (95% CI)	OR (95% CI)	OR (95% CI)	OR (95% CI)
Parental education level									
Higher education	1	1	1	1	1	1	1	1	1
Upper secondary school	1.15 (0.91–1.44)	0.97 (0.75–1.27)	1.34 (0.84–2.14)	1.13 (0.89–1.42)	0.94 (0.72–1.22)	1.23 (0.77–1.97)	1.14 (0.91–1.45)	0.94 (0.72–1.24)	1.27 (0.79–2.06)
Vocational school	1.31 (1.11–1.54)	1.02 (0.85–1.23)	1.43 (1.03–2.00)	1.27 (1.08–1.50)	0.96 (0.80–1.16)	1.28 (0.91–1.79)	1.28 (1.08–1.52)	0.95 (0.78–1.15)	1.32 (0.93–1.87)
Elementary school	1.58 (1.22–2.03)	1.34 (1.00–1.79)	1.98 (1.23–3.19)	1.52 (1.18–1.97)	1.25 (0.93–1.67)	1.71 (1.06–2.77)	1.57 (1.21–2.05)	1.25 (0.93–1.69)	1.80 (1.10–2.96)
Childhood economic difficulties									
No	1	1	1	1	1	1	1	1	1
Yes	1.34 (1.13–1.59)	1.65 (1.37–2.00)	2.62 (1.93–3.55)	1.28 (1.07–1.53)	1.65 (1.36–2.00)	2.49 (1.83–3.38)	1.17 (0.97–1.4)	1.45 (1.19–1.77)	1.86 (1.35–2.57)
Own education level									
High	1	1	1	1	1	1	1	1	1
Intermediate	1.30 (1.08–1.56)	1.25 (1.01–1.53)	1.60 (1.05–2.45)	1.24 (1.03–1.49)	1.23 (1.00–1.52)	1.52 (0.99–2.34)	1.25 (1.04–1.51)	1.23 (0.99–1.52)	1.57 (1.01–2.44)
Low	1.75 (1.45–2.11)	1.59 (1.28–1.97)	4.00 (2.68–5.96)	1.60 (1.31–1.95)	1.53 (1.22–1.92)	3.55 (2.34–5.38)	1.59 (1.29–1.95)	1.44 (1.13–1.82)	3.38 (2.18–5.24)
Occupational class									
Professional	1	1	1	1	1	1	1	1	1
Semi-professional	1.17 (0.97–1.40)	1.07 (0.87–1.31)	1.55 (1.03–2.33)	1.1 (0.91–1.32)	1.03 (0.83–1.27)	1.4 (0.92–2.12)	1.12 (0.93–1.35)	1.03 (0.83–1.28)	1.47 (0.96–2.24)
Routine non-manual employee	1.47 (1.21–1.79)	1.25 (1.00–1.57)	2.60 (1.72–3.93)	1.33 (1.09–1.63)	1.18 (0.93–1.49)	2.22 (1.45–3.41)	1.35 (1.09–1.67)	1.15 (0.91–1.47)	2.21 (1.41–3.45)
Manual worker	1.60 (1.12–2.28)	1.60 (1.09–2.36)	3.94 (2.09–7.44)	1.45 (1.01–2.09)	1.54 (1.04–2.28)	3.43 (1.80–6.56)	1.52 (1.05–2.20)	1.52 (1.01–2.27)	3.75 (1.92–7.34)
Housing tenure									
Owner occupier	1	1	1	1	1	1	1	1	1
Renter or other	1.27 (1.10–1.48)	1.07 (0.91–1.28)	1.85 (1.35–2.52)	1.23 (1.05–1.43)	1.03 (0.87–1.23)	1.68 (1.23–2.3)	1.21 (1.03–1.42)	0.97 (0.81–1.17)	1.40 (1.00–1.96)
Income level									
4th Quartile	1	1	1	1	1	1	1	1	1
3rd Quartile	1.04 (0.85–1.28)	1.11 (0.88–1.41)	1.35 (0.85–2.16)	1.02 (0.83–1.24)	1.10 (0.87–1.39)	1.29 (0.81–2.06)	1.01 (0.82–1.25)	1.06 (0.83–1.35)	1.24 (0.77–2.01)
2nd Quartile	1.25 (1.02–1.53)	1.22 (0.96–1.55)	1.48 (0.93–2.37)	1.19 (0.97–1.46)	1.19 (0.93–1.51)	1.37 (0.85–2.20)	1.20 (0.96–1.50)	1.08 (0.83–1.40)	1.26 (0.76–2.09)
1st Quartile	1.11 (0.90–1.36)	1.31 (1.04–1.66)	2.75 (1.80–4.21)	1.04 (0.85–1.28)	1.26 (1.00–1.60)	2.46 (1.60–3.78)	1.10 (0.85–1.41)	1.15 (0.87–1.54)	2.41 (1.44–4.02)
Wealth (€)									
⩾100k	1	1	1	1	1	1	1	1	1
10k–99,999	1.30 (1.08–1.57)	1.38 (1.11–1.71)	1.51 (0.98–2.33)	1.26 (1.04–1.52)	1.35 (1.08–1.68)	1.42 (0.91–2.19)	1.28 (1.06–1.56)	1.34 (1.07–1.68)	1.34 (0.85–2.09)
<10k	1.55 (1.28–1.89)	1.59 (1.27–1.99)	3.2 (2.11–4.85)	1.45 (1.19–1.77)	1.47 (1.17–1.85)	2.67 (1.75–4.07)	1.37 (1.11–1.70)	1.32 (1.03–1.69)	1.94 (1.24–3.05)
Economic difficulties									
No difficulties	1	1	1	1	1	1	1	1	1
Occasional difficulties	1.43 (1.23–1.66)	1.75 (1.47–2.09)	1.77 (1.26–2.49)	1.38 (1.18–1.61)	1.70 (1.42–2.03)	1.63 (1.16–2.29)	1.33 (1.14–1.55)	1.62 (1.35–1.94)	1.43 (1.01–2.03)
Frequent difficulties	1.79 (1.40–2.29)	2.07 (1.56–2.74)	5.44 (3.66–8.08)	1.66 (1.30–2.13)	1.91 (1.43–2.54)	4.52 (3.02–6.78)	1.44 (1.12–1.87)	1.56 (1.16–2.10)	3.07 (2.00–4.70)

CI: confidence interval; CPLD: chronic pain with low disability level; CPHD: chronic pain with high disability level; OR: odds ratio.

Reference group: no pain (OR 1.00).

Model 1: adjusted for gender and age.

Model 2: model 1 adjusted for parental education level and childhood economic difficulties.

Model 3: model 2 adjusted for immigrant background, marital status, working status, binge drinking, nicotine use, physical activity, body mass index, insomnia and mental health.

The results were similar with respect to current socioeconomic circumstances after gender and age adjustment (model 1). The respondent’s current educational level was associated with both acute and chronic pain. Individuals with low education experienced more CPHD than individuals with high education (OR 4.00, 95% CI 2.68–5.96). The association increased in magnitude with lower educational levels, which suggests a socioeconomic gradient. A similar pattern was observed for occupational class, housing tenure, wealth and economic difficulties. Income level did not show a uniform gradient in the strength of the association with pain outcomes, although belonging to the lowest income quartile was associated with both CPLD (OR 1.31, 95% CI 1.04–1.66) and CPHD (OR 2.75, 95% CI 1.80–4.21).

After adjusting for childhood SEP (model 2), the associations were slightly attenuated. In the fully adjusted model (model 3), all considered measures of SEP remained associated with pain outcomes, particularly CPHD: the strongest associations were found for low education (OR 3.38, 95% CI 2.18–5.24), manual work (OR 3.75, 95% CI 1.92–7.34) and frequent economic difficulties (OR 3.07, 95% CI 2.00–4.70). Socioeconomic disadvantage showed a stronger association with CPHD than with CPLD. In the fully adjusted model (model 3), this phenomenon was observed for childhood economic difficulties, own educational level, occupational class, household wealth and current economic difficulties.

## Discussion

### Main findings

This study of 18–39-year-old Finnish municipal employees examined the role of childhood and current SEP in acute pain, CPLD and CPHD. The main findings were, first, pain was highly prevalent already among the young employees. Second, pain was associated with all considered indicators of childhood and current SEP, and the associations remained after adjustments. Finally, we found socioeconomic disparities in the chronic pain-related disability level, and that CPHD was strongly associated with socioeconomic disadvantage. This applied to childhood and to current socioeconomic disadvantage. Participants’ own low educational level, manual work and frequent economic difficulties had the strongest associations with CPHD.

### Previous studies

To our knowledge, our study is the first to examine a broad spectrum of childhood and current SEP indicators with respect to both pain chronicity and chronic pain-related disability among young employees. Acute pain was slightly more common and chronic pain less common in our cohort compared to older Finnish employees [2]. The prevalence of chronic pain among Finnish employees is, however, higher in older age groups, which highlights the importance of identifying its early risk factors [2].

The associations of childhood and current SEP with pain were clear. Socioeconomic inequalities in the prevalence of pain were present regardless of the SEP indicator considered, although there was variation across pain outcomes and SEP indicators in the magnitude of the associations. Childhood socioeconomic disadvantage has previously been linked to pain. A Portuguese study examining intergenerational educational trajectories in pain found that a stable low or declining educational level was associated with low back pain among young women, but not men [21]. Similarly, associations have been found between childhood SEP and later low back disorders [22], as well as fibromyalgia, a condition involving chronic widespread pain [23]. Current SEP has also been associated with pain in previous studies of older employees [2]. Thus, our study confirms these earlier findings of pain being socioeconomically patterned and linked to both early life and current socioeconomic circumstances.

Noteworthy was that the more disadvantaged SEP, the stronger the association with chronic pain in general and CPHD in particular. A Swedish study proposed the concept of ‘double suffering’ when it found manual workers having both more long-term illness and experiencing illness with greater intensity and frequency [24]. Although that finding concerned illness in general, it is in line with our finding of low SEP being more strongly associated with CPHD than CPLD. We observed an indication of a two-dimensional gradient in the magnitude of the associations; the lower the SEP, the stronger the association with pain in general, with chronic pain and particularly CPHD. In parallel with this theory proposed by Blank and Diderichsen in 1996 [24], this may be viewed as a ‘double suffering from pain’ affecting individuals of lower SEP.

All our three pain outcomes showed an uneven socioeconomic distribution. Nevertheless, CPHD was consistently the pain outcome with the strongest association with socioeconomic disadvantage. We found the strongest independent associations between CPHD and a low educational level, manual work and frequent economic difficulties, which corresponds to results from previous studies [2, 7]. A Spanish population-based study found similar associations between disabling chronic pain and low educational level, manual work and low income. It did not, however, find an association between non-disabling chronic pain and SEP [25]. In line with our results, an Austrian population-based study identified a socioeconomic gradient in pain-related disability independent of pain intensity and the number of pain sites [9]. Ours, as well as these previous findings, thus indicate that the subjective level of pain-related disability may not solely be explained by pain chronicity, pain intensity and the number of pain sites, but also by other mechanisms related to socioeconomic circumstances.

Differences in health behaviours, such as alcohol use, smoking and physical activity, are factors that contribute to socioeconomic inequalities in health [12]. The prevalence of insomnia, mental ill-health and obesity are also socioeconomically patterned [18,26,27], and they might contribute to the association of SEP and pain. However, adjusting the analyses for these factors did not provide an explanation for the SEP differences in pain outcomes (Table III).

Increasing attention is being paid to life-course circumstances when examining social determinants of health. Adverse early-life exposures are known to be associated with various health outcomes in adulthood, including chronic pain [23,28]. Although the biological mechanisms that mediate the effect of childhood adversities on adulthood pain are not completely understood, existing theories suggest the involvement of the hypothalamic–pituitary–adrenal axis (HPA) and early alterations in stress responses [29]. Both physical and psychological stress mediated through the HPA axis is thought to play a central part in the pathophysiology of chronic pain [13]. Although more research is needed to unravel the mechanisms that interlink SEP and chronic pain, our study supports the need for a life-course approach for understanding the socioeconomic inequalities in chronic pain and chronic pain-related disability.

### Methodological considerations

We have comprehensively examined the relationship between socioeconomic factors and pain. Nevertheless, our study has some limitations. Although the association between socioeconomic disadvantage and pain was clear, causal inferences are unwarranted due to the cross-sectional design. In addition, our results cannot be directly applied to the general Finnish population; we did not cover employees in the private sector, and we focused on an occupational cohort. In the general population, pain can be even more prevalent. As the most disadvantaged individuals outside the labour market and on short-term working contracts were excluded, this may have contributed to a ‘healthy worker’ bias. The percentage of male respondents was low but corresponds to that of the municipal sector. The overall response rate (51.5%) was fairly low, but the non-response analysis showed that the data broadly represent the target population [14]. However, individuals with lower SEP and long-term sickness absence were somewhat overrepresented among non-respondents. Response rates in surveys have in general declined, and this is similar to other surveys. Furthermore, as respondents could only report one type of pain, we may not have captured the total individual pain burden, as multisite pain is common and acute and chronic pain can co-exist [30]. Recall bias concerning childhood SEP indicators is possible. This particularly concerns economic difficulties during childhood, which may have been only occasional. In addition, variables that are subjective (e.g. economic difficulties) and based on self-report may have been prone to reporting bias.

Our study has also several strengths. The data cover a large number of employees within all fields of work in the municipal sector. Information on pain was obtained by self-reports, which is the most appropriate way of measuring a subjective condition. We examined pain with respect to multidimensional SEP indicators from childhood to current SEP, which is necessary because different markers of SEP reflect different aspects of SEP. For example, educational level tends to remain fairly stable throughout adulthood, whereas employment status and income may fluctuate over time. The life-course perspective provides an intergenerational view on how pain is socioeconomically patterned. We also used validated and established measures, such as the CPG for assessing chronic pain-related disability [5], the Jenkins sleep questionnaire to measure insomnia and the GHQ-12 to estimate mental health [19,20].

## Conclusions

In conclusion, pain is a common complaint that contributes to disability among young employees, particularly the most socioeconomically vulnerable. Our findings suggest that both pain chronicity and the subjective level of disability due to pain follow a socioeconomic gradient. Acute pain is more and chronic pain less common among the younger employees compared to older employees. This implies that attention and interventions should be directed towards early risk factors and reasons for inequalities in pain among the young, as pain already at a young age may indicate predisposition for later chronic pain and chronic pain-related disability. More knowledge about the mechanisms interlinking socioeconomic circumstances and pain is needed.

## Supplemental Material

sj-xlsx-1-sjp-10.1177_14034948211062314 – Supplemental material for Life-course socioeconomic circumstances in acute, chronic and disabling pain among young employees: a double suffering Click here for additional data file. Supplemental material, sj-xlsx-1-sjp-10.1177_14034948211062314 for Life-course socioeconomic circumstances in acute, chronic and disabling pain among young employees: a double suffering by Pi Fagerlund, Jatta Salmela, Olli Pietiläinen, Aino Salonsalmi, Ossi Rahkonen and Tea Lallukka in Scandinavian Journal of Public Health
